# Food Safety When Eating Out—Perspectives of Young Adult Consumers in Poland and Turkey—A Pilot Study

**DOI:** 10.3390/ijerph18041884

**Published:** 2021-02-15

**Authors:** Wojciech Kolanowski, Ayse Demet Karaman, Filiz Yildiz Akgul, Katarzyna Ługowska, Joanna Trafialek

**Affiliations:** 1 Institute of Health Sciences, Faculty of Medical and Health Sciences, Siedlce University, Prusa Str. 14, PL-08-110 Siedlce, Poland; katarzyna.lugowska.zdoz@uph.edu.pl; 2 Department of Dairy Technology, Faculty of Agriculture, Aydın Adnan Menderes University, South Campus, TR-35-470 Aydın, Turkey; demet.karaman@adu.edu.tr (A.D.K.); filiz.yildiz@adu.edu.tr (F.Y.A.); 3 Department of Food Gastronomy and Food Hygiene, Institute of Human Nutrition Sciences, Warsaw University of Life Sciences (WULS), str. Nowoursynowska 166, 02-787 Warsaw, Poland; joanna_trafialek@sggw.pl

**Keywords:** eating behavior, eating out, food safety, food quality

## Abstract

Food safety is perceived differently by consumers in different countries. The objective of this study was to examine the experience of young adults regarding the safety of meals eaten outside the home in Poland and Turkey. Questionnaire surveys were conducted on a group of 400 young adults. The findings provided new insights into cross-cultural consumer perceptions of the food safety of meals eaten out. Differences in the perception of the safety of the meals eaten out concerned both the manner in which consumers chose an eating establishment, the frequency with which they ate out, their experience of the meals consumed, and their practice of lodging complaints. Consumers in Poland and Turkey experienced different problems with the health quality of meals eaten out. The experience of consumers in Turkey reflected the occurrence of numerous cases of meals of poor quality, while in Poland it was smaller. This suggests that meals eaten out in Poland (an EU country) may have a lower health risk than in Turkey (a non-EU country). The method described in this study could be an additional tool for checking the operation of food safety systems in eating out establishments.

## 1. Introduction

Eating out is becoming more common, and currently represents a substantial portion of people’s diets and household spending on food [1,2,3]. It is common, not only in EU countries but across the world [4,5]. Today’s consumption does not just mean the use of material goods and services in order to satisfy the felt needs but has also become an indicator of the standard of living, a criterion of the structure of society, and a way in which individuals communicate their identity. These are especially important for young people who become adults and in general attach great importance to social contact with friends. This is usually done out of the home, for example, spending free time together, eating together, participating in social events. However, in some countries, it does not constitute an important part of people’s everyday eating habits as it was reported in Scandinavian countries [6].

Eating out has been implicated as one of the most frequent contexts for food poisoning outbreaks [7]. The factors contributing to the risks of food-borne illness include improper cooking, improper refrigerator temperatures, and poor personal hygiene among food handlers [8]. Poor attention to food hygiene during food preparation and handling significantly increases the food safety risk of meals eaten out. Many studies have identified the critical factors driving the way consumers choose their eating establishment when eating out. These are food quality, food hygiene and safety, taste, cleanliness, staff behavior, location, reputation, and price [9,10,11,12,13,14,15,16]. However, so far, few studies have examined how consumers go about evaluating the safety level of meals that they eat outside the home. It was demonstrated that hygiene or cleanliness is more critical than quality or value and four underlying foodservice hygiene factors from the consumer perspective were identified [17]. These were food and location, staff and handling, premises and practices, and ambiance. In addition, the importance of these factors was different, for consumers with different age levels, incomes, educational attainments, and occupations [9,11,14,18]. Importantly, in many rapidly growing developing countries, the safety of meals eaten out is a significant concern [19]. Nevertheless, there is a dearth of material that examines the safety of meals eaten outside the home from the consumer perspective. In addition, there are few, if any, studies comparing the perceptions of consumers from within and outside the EU regarding food safety. Consumers eating out have no information about the preparation process, nor the origin of the ingredients, and cannot objectively evaluate the food safety level of the meals they are served [20]. The objective of this study was to examine the experience of young adult consumers regarding the safety of meals eaten outside the home. The starting hypothesis assumed that young adults from countries outside the EU would indicate different problems than those from an EU country. Two countries were selected for the study, one an EU member (Poland), and one non-member (Turkey). In both these countries, food safety standards are mandatory, although they are based on different legislation. It is obvious that due to diverse cultures and climate conditions between Poland and Turkey, the foods and nutritional preferences varied. However, till now it was not reported if general awareness and perspectives of eaten-out food safety among young adults from Poland and Turkey also varied. Young adult consumers were chosen for this research in view of their growing financial independence and because they respond to the changing environment, globalization and its impact on consumption, lifestyle, and emerging new consumer trends more intensely than other market participants.

## 2. Materials and Methods

### 2.1. Data Collection

The study was carried out in Warsaw (Poland) and Aydin (Turkey) among adult consumers aged 18–30 in Mai 2018. There were 400 participants, 200 from Poland and 200 from Turkey, 50% were men and 50% women. The group was chosen according to previous research done in Greece [21]. The participants were selected randomly. Young adults were approached in places open to the public, such as shopping malls, public facilities, or higher education institutions, and asked to complete a questionnaire. Subjects were asked about their experience and subsequent behavior when they had encountered poor quality meals and customer service while eating out. The questionnaire was a self-completed one with a given answer option to choose from. Interviewers had received training in the survey methodology and were available to assist subjects in answering the questions correctly. The survey was completely anonymous. Questionnaires were marked with an identification number and on completion were collected in a closed container [8].

The questionnaire consisted of five closed questions. The questions concerned: the frequency with which the subject ate out on weekdays and on the weekend (question 1), the most important reason governing the choice of a suitable eating establishment (question 2), the frequency with which the subject was served poor quality of meals (question 3), the frequency with which the subject complained when dissatisfied with the quality of meal (question 4), and the type of quality problems that occurred (question 5) (Table 1). Question 5, dealing with food safety, was based on Regulation (EC) No. 852/2004 of the European Parliament and of the Council of 29 April 2004 on the hygiene of foodstuffs. The other questions concerned personal impressions. Additionally, the final part of the questionnaire contained questions concerning the socio-demographic characteristics of the subjects such as age, sex, education, marital status, employment (currently), place of origin, and whether the subject lived alone or together with other people. The questionnaire was prepared in the English language, then authors from Turkey and Poland translated it into their national languages. In Turkey, the Turkish version and in Poland the Polish version was distributed among the study participants.

The questionnaire was designed by the authors of this paper based on their knowledge and experience in food safety and consumer research and a shorter version of the questionnaire used in previous research [21]. Each question was discussed, and the final version of the questions and answer variants was adopted with unanimity. Question 5 concerning food safety was based on current legislation, that is, Regulation (EC) No. 852/2004 on the hygiene of foodstuffs and Codex Alimentarius. Other questions concerned personal impressions of participants.

The reliability of the questionnaire was validated using its internal consistency. Cronbach’s alpha test was used to measure internal consistency and reliability. Cronbach’s alpha coefficient was above 0.7 in both Turkey and Poland, which indicated acceptable internal consistency.

### 2.2. Data Analysis

The results were analyzed statistically using Statistica v. 13.3 software (PL, StatSoft, Inc., Krakow, Poland). The calculations were made at a significance level of α = 0.05. The student’s *t*-test was used to compare the opinions and practices of Polish and Turkish consumers. The influence of the socio-demographic characteristics of the participants on the meal quality problems reported by consumers was tested using a one-way ANOVA. The influence of the following factors was examined: education, age, sex, marital status, employment, place of origin, and number of co-residents. If a given feature differentiated consumer indications, percentages for individual features were calculated. Cluster analysis was used to interpret the results regarding poor quality out-of-home meals and the factors dissuading consumers from eating out.

## 3. Results

### 3.1. Sociodemographic Characteristics and Overall Assessment

The sociodemographic characteristics of the participants are given in Table 2. Both groups were equal in terms of sex and age. Differentiation was found in other discriminants.

The frequency of eating out (question 1) was more similar for consumers in Poland and Turkey on weekdays than on weekends (Table 3). During the week, the largest number of consumers ate out twice (73 respondents, i.e., 36.5% in Poland, and 52 respondents, i.e., 26.0%, in Turkey), or once (64, i.e., 32.0% and 48, i.e., 24.0%, respectively). However, on weekends, in Poland, the largest number of consumers ate out once (67, i.e., 33.5%) or twice (56, i.e., 28.0%), while in Turkey, 80 respondents, i.e., 40% of the research group ate out only sometimes.

* not significant.

**Figure 1:**
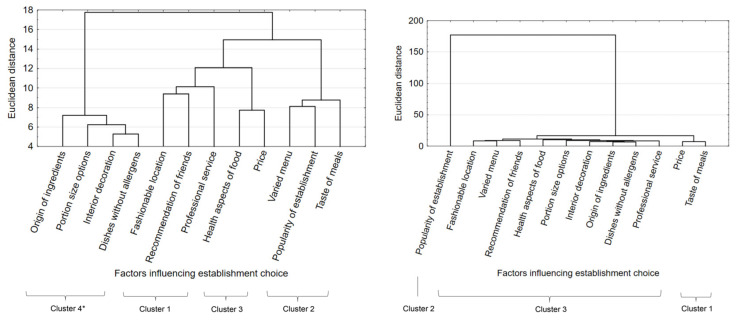
Factors influencing the choice of a place to eat out at: (**a**) in Poland, (**b**) in Turkey. * Clusters numbered according to their importance to consumers.

The factors identified in the questionnaire were grouped (question 2) taking into account similarities and frequency of indications. To perform the grouping, multidimensional cluster analysis was used; the results are shown in Figure 1.

In Poland, four groups of factors were identified, and three groups could be distinguished in Turkey. Consumers in Poland were most influenced by a combination of factors such as location, recommendations, and customer service (cluster 1: 59.0%). In second place of importance were a varied menu, the popularity of the venue, and the taste of the meal (cluster 2: 143%); and in third place were the price and the health aspect of meals (cluster 3: 92.0%). Factors grouped in cluster 1 made little impact on the choice of location for eating out (45.0%). However, in Turkey when consumers were choosing an establishment to eat out at, the most important factors were both price and taste (cluster 1, 80.0%). The popularity of the establishment took second place (cluster 2: 73.0%). On the other hand, a group of several factors, such as professional service, health aspects, dishes without allergens, recommendation of friends, varied menu, interior decoration, the origin of ingredients, availability of differently sized portions, fashionable location (cluster 3: 30.5%), had the smallest impact.

### 3.2. Consumer Experience and Practice Regarding Meals of Inadequate Health Quality

The study looked at whether consumers, when going out to eat, were served poor quality meals and, if this was the case, what their complaint practices were (questions 3 and 4). Consumers from both countries noticed poor quality meals served outside the home at a similar frequency (Table 4). In both countries, the largest number of consumers indicated that this occurred from time to time (Poland 96 respondents, i.e., 48.0%, Turkey 89 respondents, i.e., 44.5%). However, the practice of making complaints was different in both countries. In Turkey, significantly more consumers than in Poland complained very often (Turkey 63 respondents, i.e., 31.5%, Poland 22 respondents, i.e., 11.0%). The largest group of consumers from both countries made complaints from time to time. However, significantly more consumers complained in Poland (86 respondents, i.e., 43.0%) than in Turkey, where 48 respondents, that is, 24.0% complained hardly ever.

The study also looked at what problems consumers in both countries had encountered as regards the food safety aspects of meals eaten outside the home (question 5). To this end, several statistical analyses were carried out. As a first step, the indicated problems were grouped using multidimensional cluster analysis, then any differences in the indicated problems were examined using the student’s *t*-test and, finally, ANOVA was used to determine whether the sociodemographic characteristics of consumers differentiated the indicated problems.

**Figure 2:**
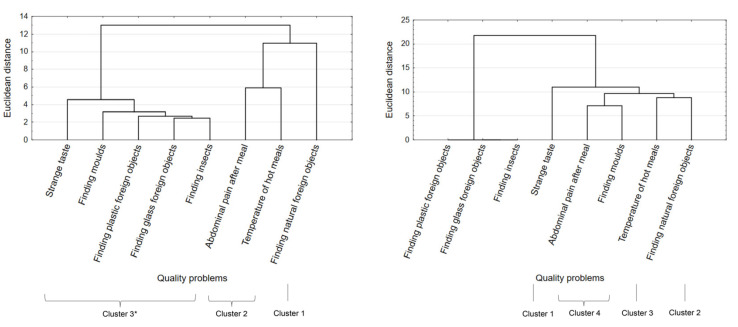
Food safety problems encountered when eating out: (**a**) in Poland, (**b**) in Turkey. * Clusters numbered according to their importance to consumers.

Multidimensional cluster analysis was employed to group the problems indicated by consumers based on the similarities, and the frequencies, of their indication. In Poland, three clusters were identified, and four in Turkey (Figure 2). In Poland, the most serious problem was finding natural foreign objects, such as stones, seeds, pieces of bone (Poland cluster no. 1, 40.0%), while in Turkey it was strange taste (cluster 1: 78.5%). In joint second position in Poland were abdominal pain after a meal, and hot meals being served at too low a temperature (cluster 2: 20.75%), while in Turkey in second position was finding natural foreign objects (cluster 2: 52.5%). In Poland, the least important factors were strange taste; finding molds, glass or plastic foreign objects, and insects in a meal (cluster 3: 4.4%). In Turkey hot meals being served at too low a temperature was in third place (cluster 3: 35.5%), and in joint fourth place, abdominal pain after meal, and finding molds (cluster 4: 33.0%). Turkish consumers did not indicate any problems at all with glass and plastic foreign objects, or insects. The next step in the statistical analysis looked at the significance of the differences between the quantities of the specific problems with the quality of meals eaten outside the home. This was performed using the student’s *t*-test (Table 5). The quality problems that were more frequent in Poland and those that appeared more frequently in Turkey were identified. The calculations show that generally, problems with the quality of meals eaten away from home arose more often in Turkey than in Poland. The exceptions were problems that did not occur in Turkey at all, in particular finding the presence of insects, pieces of glass, and plastic.

The next step was to check whether the sociodemographic characteristics of consumers differentiated the indications of problems with respect to the quality of meals eaten outside the home (Table 6). The results of ANOVA calculations lead to the conclusion that the examined characteristics of Turkish consumers had only a minimal impact on the nature of the indications of problems with the food safety aspects of meals eaten out. Correlations were only found in the cases of strange taste as well as finding molds (Table 6). With respect to strange taste, consumer indications showed a correlation with marital status and place of origin, while in the case of finding molds, the correlation was with the educational attainments of consumers.

However, the impact of the examined circumstances on the indications of Polish consumers was more noticeable. Many factors influenced the indications of individual problems with the quality of meals eaten outside the home. Most often it was the level of education, the sex of consumers, their marital status, and the size of their home. Better educated consumers indicated more problems associated with the presence of insects, strange taste, and fragments of glass. Problems with natural foreign objects, and hot meals being served at too low a temperature, were most often noticed by consumers living in families of up to four people. Consumers who found fragments of glass in meals were exclusively singles. Consumers living in big cities experienced problems with natural foreign bodies and glass.

## 4. Discussion

This is the first study to compare the perception of consumers in Poland and Turkey of the safety of eating food outside the home. Young adults from Poland and Turkey exhibit different eating out behaviors with respect to frequency. In Poland, they are more likely than in Turkey to eat out as frequently on weekdays as on weekends. Consumers in Poland ate out as frequently, or slightly more frequently, than those in other EU countries [3,5,6,22]. In a number of countries, although governments or institutions do provide advice on how to choose more healthy diet options when eating out, they do not give any guidance on the food safety and quality aspects [23].

Research conducted among young consumers in Poland and Turkey revealed their different approaches in choosing places to eat out at. In addition, their choices were based on different criteria than those previously described in the literature on the subject. For example, when choosing a restaurant in Korea, consumers were most influenced by the freshness of the food, followed by its taste, the hospitality they received, and the degree of cleanliness maintained by the establishment [16]. The characteristic of dishes was the most important factor in China [24], while in the United States the key factors were outstanding quality value and practical value [15,25], and in Delhi (India) the significant deciders were family preferences, habits and perceptions learned in childhood, convenience, and food safety and health [26]. Other authors have pointed out that word-of-mouth recommendations, external ratings [14], the degree of crowding, and review ratings [13] can all affect the choice of eating establishment. Young consumers in Poland and Turkey used different criteria when choosing places to eat out at. Although it is possible to find similarities to the results of other authors’ research on the quality of meals [9,12,26], cluster analysis revealed different sets of factors affecting their decisions. For example, in Poland, the health aspect was taken into account along with the price of meals, while in Turkey, equally important were the price of meals and their taste. This may be the result of a different way of perceiving the quality of meals, or the absence of relevant consumer awareness. In Turkey, the price of meals was of great importance, as it was also in the United States [27] and in China [24]. It was reported that only in top restaurants the customer care was the most important, and price the least important attribute [10]. However, among young Polish consumers, price did not play such a large role in the choice of places to eat out at as in other countries, which may indicate their improved economic circumstances. In addition, many studies showed that the relative importance of the factors changed, mainly depending on the consumer’s age, income, education, and occupation [9,11,14,18].

Food safety is one of the credibility attributes of food [25,26,28]. Nevertheless, the opinions of consumers regarding the safety of meals eaten out are treated only fleetingly in the literature on the subject. There are no comprehensive studies describing the experience of consumers regarding the safety of meals eaten outside the home. Typically, such studies only deal with quite narrow aspects of food safety [20,29]. Taking into account the fact that consumers might not necessarily understand the concept of “food safety”, this study used straightforward descriptions of the safety problems that could occur with meals eaten outside the home. They covered: the occurrence of the symptoms of food poisoning, the discovery of foreign bodies, and the serving of hot meals at too low a temperature. These circumstances are quite visible and easily recognizable and by utilizing them, consumers can easily make assessments as to the level of food safety. The most frequently found foreign bodies were pests, glass, and metals [30,31]. In addition, the presence of plastic and natural foreign bodies, such as fragments of bones, stems, and stones, was examined. Reports of their occurrence was a common feature among indications from consumers in Poland and Turkey. Natural foreign bodies were indicated “most often” in Poland and “very often” in Turkey. In comparison, such problems very rarely occurred in Great Britain [32]. Other than this common feature, young consumers in Poland and Turkey indicated different problems with the safety of meals eaten outside the home. Cluster analysis calculations highlighted these differences. Indications regarding the presence of foreign bodies and the occurrence of typical symptoms of food poisoning, such as abdominal pain, prevailed in Poland [33] in combination with hot meals being served at too low a temperature. In contrast, in Turkey the largest proportion of consumers associated problems with the safety of meals only with strange taste, which is not an adequate method of ensuring safety, though the smallest group of consumers did correctly associate abdominal pain or finding molds as signs of poor food safety. Mold in food also occurred in Brazil [34], and sporadically appeared in Polish meals eaten out. The reports from the USA showed overall poor knowledge of safe food practices among students [35]. However, other research showed that food safety education promotes more optimal food safety behavior [36]. This indicates the importance and common inadequacy of food safety education at younger ages.

Examination of the significance of the occurrence of food safety problems highlights the fact that all the problems were more often indicated by consumers in Turkey than in Poland. The biggest differences concerned two problems, strange taste and finding molds. This may indicate two non-exclusive causes: better food safety quality of meals eaten out in Poland, and/or greater scrutiny of such meals by consumers in Turkey. On the basis of information in the literature on the subject, it can be concluded that consumers with different cultural backgrounds perceive the safety of eaten out meals differently. For example, in China, the vast majority of consumers paid attention to unhygienic food [24], and in Great Britain to the cleanliness of premises, cutlery, and glasses [32].

This study shows that the frequency with which young adults in Poland and Turkey noticed poor quality meals was similar. However, consumers from Turkey made complaints more often than in Poland. This could be the result of the different cultural backgrounds of the two groups. Other authors have stated that there is no universal pattern of consumer complaint behavior [37]. The authors pointed out that consumers from different cultures have different needs and expectations when they complain. Examination of socio-demographic factors differentiated that problems with the quality of meals eaten out of the home occur much more often in Poland than in Turkey. This confirms the presence in Turkey of other mechanisms that affect the perception of the health safety of such meals. Published research regarding complaints studies indicates that complaints about food are becoming more common. This applies to both food itself (e.g., boxes of chocolate, bread, cheese, pizzas, milk), as well as the production and eating facilities such as hotels, restaurants, and community services [34,38,39]. However, the practice of complaining about poor quality food varies greatly, that is, from around 30% of respondents in England [32] to 82% in Brazil [34]. Few studies have looked at consumer complaints occasioned by a specific cause [38]. According to the authors, the most frequently reported hazards in Brazilian dairy foods were the presence of foreign objects (42.4%), insects (23.3%), hair (15.2%), plastics (11.1%), metal (6.2%), and fabric (1.8%). Comparing these results with data obtained in this study from young adults in Poland and Turkey indicates that consumers do pay attention to the presence of foreign bodies in food products, and the most probable reason is the ease with which their presence can be recognized.

Analysis of empirical data concerning eating out, and the demographic factors which can influence consumer preferences and behavior, has become increasingly important, not only for consumers and food providers but also for policymakers and investors. Policy incentives are being introduced to increase spending in the hospitality industry, to increase employment, and to encourage greater awareness of food safety and public health security [39]. Understanding consumer perception and behavior is an important prerequisite to minimize the safety risks. The cultural and geographical background of consumers plays a significant role in determining their acceptance of and reaction to foods, and this point is generally neglected in the literature [40,41]. Although consumer complaints and comments are usually considered a negative reflection of the business, such comments can provide useful information for authorities and hospitality industry investors that they could use to their advantage [39]. Therefore, this study is of significant importance not only for the producers and consumers of meals served out of the home but also for the institutions responsible for the supervision of food production and delivery. This study has certain limitations in terms of both its methodology and its applicability. It only included young adults from one city in each country, so the current findings may not be applicable to the whole population of young adults in Poland and Turkey. In addition, the study was conducted only in Warsaw and Aydin. Consumer perception may be different in other places. In order to obtain a more comprehensive characterization of consumer experiences and behavior with respect to the low quality of meals when eating out, further research should be carried out. It is advisable to study larger population groups in different age ranges in combination with an analysis of the appropriate socio-demographic factors. Moreover, the questionnaire should be enlarged and include questions regarding the kind of food consumed and the reasons for eating out of the home.

Another limitation shared with other studies [42] is that the method did not include any microbiological analysis of the meals eaten out. However, it would be difficult to combine such an analysis with an investigation of the opinions of consumers on the safety aspects of meals eaten out. Putting such limitations aside, our findings do suggest the need for an established verification methodology for the safety of meals eaten out.

## 5. Conclusions

The findings provide new insights into cross-cultural consumer perceptions of the food safety of meals eaten out. Differences in the perception of the food safety of the meals eaten out by consumers from the EU, and outside the EU, concerned both the manner in which consumers chose an eating establishment, the frequency with which they ate out, their experience of the meals consumed, and their practice of lodging complaints were found. Consumers in Poland and Turkey experienced different problems with the health quality of meals eaten out. The experience of consumers in Turkey reflected the occurrence of numerous cases of meals of poor quality, while the number of such instances in Poland was smaller. This suggests that meals eaten away from home consumed in Poland (an EU country) may have a lower health risk than in Turkey (a non-EU country). The verification method for the safety of meals eaten out described in this study could be an effective method of checking the operation of food safety systems used by the producers and distributors of meals that are eaten outside the home. The findings could help guide the relevant authorities in designing public health campaigns and other interventions to promote healthier and safer eating out.

## Figures and Tables

**Table 1:** The questionnaire.

No.	Question/Options of the Answer	
1.	How often do you eat out?—Mark one answer in each column.	
	On weekdays (from Monday till Friday)	At the weekend
	□ hardly ever	□ hardly ever
	□ once	□ from time to time
	□ twice	□ once
	□ more than 3 times	□ twice
	□ every day	□ more than 3 times
2.	What is most important to you when choosing a place to eat out at?—You can mark several answers.	
	□ taste of meals	□ recommendation of friends
	□ price	□ a varied menu
	□ professional service	□ interior decoration
	□ popularity of the establishment	□ the origin of ingredients
	□ health aspects of food	□ the availability of differently sized portions
	□ availability of dishes without allergens	□ fashionable location of the establishment
	□ hot sandwich, baguette, casserole	
3.	How often have you been served a poor the quality of your meal been poor?—Mark one answer.	
	□ very often	□ from time to time
	□ hardly ever	□ never
4.	How often do you complain when the quality of your meal has been poor?—Mark one answer.	
	□ very often	□ from time to time
	□ hardly ever	□ never
5.	What kind of quality problems do you find the most often in meals you have bought, if any?—You can mark several answers	
	□ finding natural foreign objects (stones, seeds, pieces of bone)	□ strange taste
	□ meal was not sufficiently hot	□ finding molds
	□ finding insects	□ experiencing abdominal pain after a meal
	□ finding glass foreign objects	□ finding plastic foreign objects

**Table 2:** The socio-demographic characteristics of participants.

Factors	Variants	Poland (%)	Turkey (%)
Age	18–24 years old	51.5	55.0
	25–30	48.5	45.0
Sex	Female	50.0	50.0
	Male	50.0	50.0
Education	Secondary	45.5	21.5
	Higher	54.5	78.5
Marital/Family Status	Single	49.0	62.0
	Family up to 4 persons	32.5	34.0
	Family above 4 persons	18.5	4.0
Employment (currently)	Yes	64.5	70.0
	No	35.5	30.0
Place of Origin	Small town or village	30.0	4.0
	Medium city	28.0	44.0
	Big city	42.0	52.0
Co-Residents in Household	Living alone	28.0	38.5
	With a partner	26.5	14.0
	With family/friends up to 4 persons	24.5	30.5
	With family/friends above 4 persons	21.0	11.5

**Table 3:** The frequency of eating out in Poland and Turkey.

Frequency of Eating Out from Monday to Friday	Poland (%)	Turkey (%)	Student’s *t*-Test *p*-Value	Frequency of Eating Out at the Weekend	Poland (%)	Turkey (%)	Student’s *t*-Test *p*-Value
hardly ever	16.5	22.0	ns *	hardly ever	13.0	14.0	ns *
once	32.0	24.0	ns *	sometimes	23.5	40.0	0.000
twice	36.5	26.0	0.030	once	33.5	19.0	0.000
more than 3 times	15.5	19.0	ns *	twice	28.0	18.0	0.017
every day	0.0	9.5	0.000	more than 3 times	2.0	8.0	0.005

**Table 4:** Consumer experience of poor-quality meals and the practice of making complaints.

Poor-Quality Meals Experience	Being Served a Poor-Quality Meal (Question 3)			Complaining When Served a Poor-Quality Meal (Question 4)		
	Poland (%)	Turkey (%)	T Student *p*-Value	Poland (%)	Turkey (%)	T Student *p*-Value
Very often	0.0	4.0	0.002	11.0	31.5	0.000
Hardly ever	28.0	23.0	ns *	43.0	24.0	0.000
From time to time	48.0	44.5	ns *	42.5	40.0	ns *
Never	24.0	28.0	ns *	3.5	4.0	ns *

* not significant.

**Table 5:** Quality problems reported by consumers in Poland and Turkey.

Kind of Problem	Poland (%)	Turkey (%)	Student’s *t*-Test *p*-Value
Finding insects	5.0	0.0	0.001
Finding glass foreign objects	3.0	0.0	0.013
Finding plastic foreign objects	4.0	0.0	0.000
Finding natural foreign objects	40.0	52.5	0.012
Temperature of hot meal	19.5	35.5	0.000
Strange taste	5.0	78.5	0.000
Finding molds	5.0	34.5	0.000
Abdominal pain after a meal	22.0	31.5	0.031

**Table 6:** Factors which influenced reporting of meal quality problems by consumers.

Kind of Quality Problems	Country	Education (%)	Age (%)	Sex (%)	Marital Status (%)	Employment (%)	Place of Origin (%)	Inhabitation (%)
Finding natural foreign objects	Poland	ns *	ns *	*p* = 0.000 F 27.5 M 72.5	*p* = 0.000 S 48.3 F4 28.5 Fa4 23.2	ns *	*p* = 0.000 S 34.0 M 21.4 B 44.6	*p* = 0.000 A 28.5 B 32.3 C 16.0 D 23.2.0
	Turkey	ns *	ns *	ns *	ns *	ns *	ns *	ns *
Inadequate temperature of hot meal	Poland	ns *	ns *	*p* = 0.001 F 71.8 M 28.2	*p* = 0.001 S 36.0 F4 56.4 Fa4 7.6	*p* = 0.020 E 79.5 nE 20.5	ns *	ns *
	Turkey	ns *	ns *	ns *	ns *	ns *	ns *	ns *
Finding insects	Poland	*p* = 0.020 S 10.0 H 90.0	ns *	*p* = 0.048 F 80.0 M 20.0	*p* = 0.020 S 90.0 F4 0.0 Fa4 10.0	ns *	ns *	ns *
	Turkey	**	–	–	–	–	–	–
Strange taste	Poland	*p* = 0.002 S 0.0 H 100	ns *	ns *	ns *	ns *	ns *	ns *
	Turkey	ns *	ns *	ns *	*p* = 0.021 S 66.3 F4 31.2 Fa4 2.5	ns *	0.003 S 7.0 M 38.8 B 54.2	ns *
Finding molds	Poland	ns *	ns *	ns *	ns *	ns *	ns *	ns *
	Turkey	*p* = 0.013 S 11.6 H 88.4	ns *	ns *	ns *	ns *	ns *	ns *
Abdominal pain after a meal	Poland	ns *	*p* = 0.012 A 68.2 B 31.8	ns *	ns *	ns *	*p* = 0.006 S 47.8 M 13.6 B 38.6	ns *
	Turkey	ns *	ns *	ns *	ns *	ns *	ns *	ns *
Finding glass foreign objects	Poland	*p* = 0.023 S 0.0 H 100.0	ns *	ns *	*p* = 0.034 S 100.0 F4 0.0 Fa4 0.0	ns *	*p* = 0.014 S 0.0 M 0.0 B 100.0	ns *
	Turkey	–	–	–	–	–	–	–
Finding plastic foreign objects	Poland	ns *	ns *	ns *	ns *	ns *	ns *	ns *
	Turkey	–	–	–	–	–	–	–

* not significant ** problems do not occur. Education: secondary (S), higher education (H); Age: 18–24 (A), 25–30 (B); Sex: female (F), male (M); Marital status: single (S), family up to four persons (F4), family above four persons (Fa4); Employment: employed (E), unemployed (nE); Place of origin: small town (S), medium city (M), big city (B); Inhabitation: living alone (A), as a couple (B), with family/friends up to four persons (C), with family/friends above four persons (D).

## Data Availability

The data presented in this study are available on request from the corresponding author.
